# Association between statins and progression of osteoarthritis features on magnetic resonance imaging in a predominantly pre-radiographic cohort: the Vancouver Longitudinal Study of Early Knee Osteoarthritis (VALSEKO): a cohort study

**DOI:** 10.1186/s12891-022-05900-x

**Published:** 2022-10-28

**Authors:** Jagdeep Gill, Eric C. Sayre, Ali Guermazi, Savvas Nicolaou, Jolanda Cibere

**Affiliations:** 1 Faculty of Medicine, University of British Columbia, Vancouver, BC Canada; 2 Arthritis Research Canada, Vancouver, BC Canada; 3 Department of Radiology, VA Boston Healthcare System, Boston University School of Medicine, Boston, MA USA; 4 Vancouver Coastal Health, Faculty of Medicine, University of British Columbia, Vancouver, BC Canada

**Keywords:** Osteoarthritis, Statins, Cartilage, Magnetic resonance imaging, Diagnostic imaging

## Abstract

**Background:**

To evaluate the effect of statin use on osteoarthritis (OA) incidence/progression using magnetic resonance imaging (MRI) in a population-based cohort with predominantly pre-radiographic knee OA.

**Methods:**

A cohort aged 40–79 years with knee pain was recruited using random population sampling and followed for 7 years. Baseline exclusions were inflammatory arthritis, recent knee surgery/injury, and inability to undergo MRI. At baseline, current statin use was ascertained. Baseline and follow-up MRIs were read semi-quantitatively for cartilage damage (grade 0–4, 0/1 collapsed, 6 regions), osteophytes (grade 0–3, 8 regions), bone marrow lesions (BML) (grade 0–3, 6 regions) and effusion (grade 0–3). The primary outcome was cartilage damage incidence/progression, while secondary outcomes were incidence/progression of osteophytes, BML, and effusion, each defined as an increase by ≥1 grade at any region. To ensure population representative samples, sample weights were used. Logistic regression was used to assess the association of statin use at baseline with incidence/progression of MRI outcomes. Analyses were adjusted for sex, age, BMI, and multiple comorbidities requiring statin therapy.

**Results:**

Of 255 participants evaluated at baseline, 122 completed the 7-year follow-up. Statin use was not significantly associated with progression of cartilage damage (OR 0.82; 95% CI 0.17, 4.06), osteophytes (OR 3.48; 95% CI 0.40, 30.31), BML (OR 0.61; 95% CI 0.12, 3.02), or effusion (OR 2.38; 95% CI 0.42, 13.63), after adjusting for confounders.

**Conclusion:**

In this population-based cohort of predominantly pre-radiographic knee OA, statins did not affect MRI incidence/progression of cartilage damage, BML, osteophytes or effusion. Therefore, statin use does not appear to affect people with pre-radiographic stages of knee OA.

## Key points

1. Our study assessed the effect of statin use in a predominantly pre-radiographic knee osteoarthritis cohort
2. We assessed osteoarthritis incidence/progression using MRI imaging which is more accurate than knee radiographs
3. Statin use does not affect MRI incidence/progression of knee osteoarthritis in our cohort

## Background

Hip and knee osteoarthritis (OA) are ranked the 11th highest contributor of global disability [1]. One Canadian study found the prevalence of OA to be 14.2% in patients over 30 years of age [2]. Currently, the pharmacological management of OA can only reduce pain with the use of nonsteroidal anti-inflammatory drugs (NSAIDs) and corticosteroid injections and there are no medical therapies that can halt or slow down the progression of OA.

Statins have traditionally been used to lower cholesterol and prevent stroke and myocardial infarctions. However, recently there has been interest in the effect of statins on OA progression. An in-vitro study by Yudoh et al [3] found that statins inhibit the effects of interleukin-1B (IL-1B), a pro-inflammatory cytokine. These effects refer to reducing IL-1B induced matrix metalloproteinases (MMP) production, increasing proteoglycan production, and extending the life span of chondrocytes that was initially reduced by IL-1B. Yudoh et al. [3] also found that statins reduced cartilage degeneration in osteoarthritis mice models. Simopoulou et al [4] reported that atorvastatin reduced IL-1B production, MMP production, and increased collagen and aggrecan production in osteoarthritic chondrocyte cultures. It is well understood that inflammation plays a role in the progression and symptoms of OA [5, 6], and in a review article by Conaghan et al., statins were described to reduce levels of inflammatory cytokines and T cells, inhibit leukocyte endothelial adhesion, and reduce nitric oxide (NO) synthase all of which leads to reduced inflammation [5]. Statins have also been shown to reduce osteoclast function and increase osteoblast activity in subchondral bone; an area affected by osteoarthritis [5]. Lastly, since hypertension and ischemia can worsen osteoarthritis progression, the beneficial anti-atheromatous effect of statins may slow progression [5]. In contrast to these potentially beneficial effects, other literature found that statins increase nitric oxide production which would inhibit cartilage matrix synthesis, shorten chondrocyte lifespan, and increase cartilage breakdown [7, 8].

There are several clinical studies that have evaluated the effect of statins on OA progression with some reporting statins to be harmful [9] or protective [10–12], while others did not find any correlation between statin usage and OA progression [13, 14]. All of these studies used radiographs as the primary outcome measure. Although radiography is useful in the assessment of osteoarthritis progression, it uses joint space narrowing (JSN) as an indirect measure of cartilage damage progression and thus is unable to directly measure articular cartilage damage. In contrast, MRI can detect early cartilage damage over a short time interval and has the advantage of being able to evaluate other tissue abnormalities that are involved in the OA disease process, such as bone marrow lesions (BML) and effusion [15]. To the best of our knowledge, there are no studies that have assessed the effect of statin use on cartilage damage progression or other MRI outcomes of OA progression. The goal of this cohort study was to evaluate the association between statin use and incidence/progression of cartilage damage, effusion, osteophytes, and bone marrow lesions on MRI in a population-based cohort of participants with predominantly pre-radiographic OA followed over 7 years.

## Methods

### Population

This study used data from the Vancouver Longitudinal Study of Early Knee Osteoarthritis (VALSEKO), a population-based cohort study with 7-year follow-up [16]. At baseline, participants 40–79 years old were recruited using random population sampling of the Greater Vancouver Area. Subjects were included if they had “pain, aching or discomfort in or around the knee on most days of the month at any time in the past” and “any pain, aching or discomfort in or around the knee in the last 12 months”. Participants were excluded if they had MRI contraindications, inflammatory arthritis or fibromyalgia, knee arthroplasty, knee trauma or surgery in the past 6 months, or knee pain referred from the hips or back [17].

**Fig. 1:**
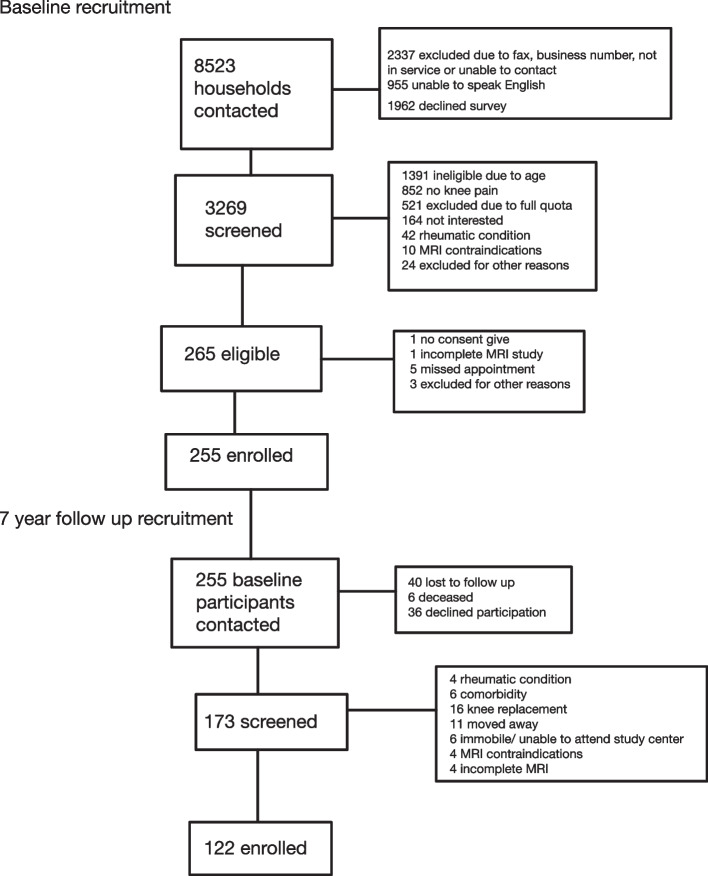
Flow diagram of cohort recruitment at baseline and at 7 year follow up. The boxes to the right list reasons for exclusion at each step [16]

Recruitment has been described in detail previously [17] and is described in Fig. 1. During recruitment, a list of households was obtained from the telephone directory listings. This list was used to mail invitation letters to randomly selected households. Age-sex strata were used to ensure adequate sample size across the age-sex spectrum with capping at 35 participants for each stratum. Participants underwent an initial standardized telephone screening for eligibility followed by a detailed in-person eligibility screen. Of 8523 contacts, 3269 households agreed to be screened and spoke English. Of these, 42.6% were excluded due to age, 26.1% did not have knee pain, 15.9% due to full age-sex stratum, 5% due to inability to attend the study center, 1.3% due to inflammatory arthritis and 1% had MRI contraindications.

At the 7 year follow up, all participants were invited to return to the study center. Exclusions at follow-up were MRI contraindications, inflammatory arthritis or fibromyalgia, knee arthroplasty, and inability to attend the study center.

### Clinical assessment

At baseline, participants completed detailed self-administered questionnaires on knee symptoms and general health. Current statin use and dosage was obtained at baseline by having participants list all current medications, dose, and duration. The following question was used to ascertain statin and other medications use: *“Please list all current medications that you are taking below, including the dose or how much of the medication you take, and how often you take it (for example NAME: Glucosamine, DOSE: 500mg, HOW OFTEN: twice a day). Please note that the types of medications have been divided into prescribed, over the counter, herbal therapies/ supplements, and vitamins and minerals.”* In order for participants to provide accurate information on their current medications, we asked participants to bring their medication and supplement bottles with them to the assessment visit or to bring a list of their medications/supplements. This information was then reviewed by the principal investigator, who confirmed all medication use and dosage. If a participant forgot to bring the medication list, this information was obtained by the research assistant either at the subsequent MRI visit or by telephone follow-up and this information was reviewed by the principal investigator for completeness. Self-reported height and weight were used to calculate body mass index (BMI) (kg/m^2^). Participants completed the Western Ontario and McMaster Universities (WOMAC) Osteoarthritis Index version VA 3.1 which assesses for pain, stiffness, and function. The subscales were normalized to a scale from 0 to 100 with a higher grade indicating worse pain, stiffness, and function.

### Radiographic assessment

At baseline, subjects had weight-bearing posteroanterior knee radiographs done using the fixed flexion technique with the SynaFlexer positioning frame [17, 18]. The radiographs were scored using the Kellgren Lawrence (KL) scale (0–4) by two independent readers (JC and SN) with good interrater reliability and an intraclass correlation coefficient of 0.79 [19]. The two readers were blinded to clinical and MRI assessment of the participants. Reading differences were resolved by consensus [17].

### MRI assessment

Participants underwent MRI of the knee at baseline and at seven-year follow-up. The MRI was obtained on a GE 1.5 T magnet using a transmit-receive extremity knee coil. All MRIs were performed at a single center. The MRI imaging protocol included four sequences described previously [17]:

- T1-weighted fat-suppressed 3-dimensional spoiled gradient echo (SPGE) sequence in a steady state sequence, with images obtained in the sagittal plane and reformat images in the axial and coronal planes
- fat-suppressed T2-weighted fast spin-echo (FSE) sequence, with images obtained in the coronal plane
- T1-weighted FSE sequence, with images obtained in the oblique sagittal plane (angulated to according to the course of the anterior cruciate ligament)
- T2-weighted FSE sequence, with images obtained in the oblique sagittal plane (angulated to according to the course of the anterior cruciate ligament)

The scoring system used in our study has been previously described by Disler et al [20]. We did not use any of the more recent grading systems such as MOAKS since these grading systems did not exist at the time this study was recruiting participants and obtaining baseline MRIs. Cartilage damage was graded on a scale from 0 to 4, where 0 = normal, 1 = abnormal signal without a cartilage contour defect, 2 = contour defect of < 50% cartilage thickness, 3 = contour defect of 50–99% cartilage thickness, and 4 = contour defect of 100% cartilage thickness with a subjacent bone signal abnormality [17]. We combined cartilage grades 0 and 1, since a grade 1 lesion is of indeterminate significance and there were very few participants with grade 1 cartilage defect. Bone marrow lesions (BML) were graded on a 0–3 scale, where 0 = no bone marrow lesion, 1 = mild lesion (< 25% of the site), 2 = moderate lesion (25–49% of the site), and 3 = large lesion (≥ 50% of the site) [16, 17]. We assessed six areas of the knee joint for cartilage damage and BML: the medial and lateral tibial plateaus, the medial and lateral femoral condyles, the patella, and trochlear groove. Osteophyte formation was graded on a scale from 0 to 3 where 0 = no osteophyte, 1 = small beak like osteophyte, 2 = intermediate sized osteophyte, and 3 = proliferative or mushroom sized osteophytes. We assessed eight areas of the knee joint for osteophytes: medial and lateral tibia, the medial and lateral femur, and the superior, inferior, medial, and lateral patella. Lastly, knee effusion was graded on a scale from 0 to 3, where 0 = no effusion, 1 = mild effusion, 2 = moderate effusion, and 3 = large effusion. Due to the small cohort size, incidence and progression could not be evaluated separately. Therefore, any change in score by ≥1 grade at any region was considered incidence/progression for cartilage, osteophytes, BML, and effusion. Baseline and follow up MRIs were read semi-quantitatively by an experienced musculoskeletal radiologist (AG) side-by-side. The reader was blinded to clinical and radiographic information as well as time sequence. The intraclass correlation coefficients for each parameter are as follows: cartilage 0.84–1.00, osteophytes 0.77–0.89, BML 0.81–0.93, and effusion 0.76 [16].

All study participants provided written informed consent. The study received ethics approval from the Clinical Research Ethics Board, University of British Columbia and was conducted in accordance with the declaration of Helsinki. (Ethics ID: H09–02046).

### Statistical analysis

The baseline descriptive characteristics were summarized using frequencies or means +/− standard deviation (SD) in Table 1. Logistic regression models were developed to evaluate the association between statin use and incidence/progression of cartilage damage, osteophytes, BML and effusion. The analysis was adjusted for age, sex, and BMI. We also adjusted for hypertension, dyslipidemia, cardiovascular disease, cerebrovascular disease, and diabetes since these comorbidities are frequently associated with statin use. To adjust for these comorbidities, we combined all these variables into a propensity score. Since our cohort had few participants with cardiovascular disease and cerebrovascular disease, these two comorbidities were treated as a single variable. Because the distribution of KL grade differed significantly between the statin and non-statin users, we performed a sensitivity analysis adjusting for KL grade in addition to the above variables.

**Table 1:** Baseline characteristics, *N* = 122, n (%) unless otherwise specified

	Overall (wgt. *N* = 122.0) n (%) or mean (SD)	Statin Users (wgt. *N* = 9.4^a^) n (%) or mean (SD)	Statin Non-users (wgt. *N* = 112.6) n (%) or mean (SD)	*p*-value**
Age, mean (SD)	55.5 (9.1)	61.7 (8.9)	55.0 (9.0)	0.031
Female	68.0 (55.7)	3.8 (40.9)	64.2 (57.0)	0.340
BMI, mean (SD)	26.1 (4.0)	29.5 (4.2)	25.8 (3.9)	0.007
Hypertension	18.9 (15.5)	2.6 (27.5)	16.4 (14.5)	0.293
Dyslipidemia	15.9 (13.1)	6.2 (66.1)	9.7 (8.7)	< 0.001
Cardio/cerebrovascular disease	5.0 (4.1)	1.9 (20.5)	3.1 (2.8)	0.009
Diabetes Mellitus	10.4 (8.5)	3.6 (38.9)	6.8 (6.0)	< 0.001
KL grade				< 0.001
0	49.7 (40.8)	0.8 (8.1)	49.0 (43.5)	
1	24.0 (19.7)	1.1 (11.3)	23.0 (20.4)	
2	27.4 (22.5)	0.9 (9.2)	26.6 (23.6)	
3	13.8 (11.3)	4.7 (49.6)	9.1 (8.1)	
4	7.1 (5.8)	2.0 (21.8)	5.0 (4.5)	
WOMAC pain, mean (SD)	19.1 (17.3)	24.7 (21.2)	18.6 (17.0)	0.304
WOMAC function, mean (SD)	16.8 (17.3)	25.5 (17.5)	16.1 (17.2)	0.111
WOMAC stiffness, mean (SD)	23.3 (23.0)	45.7 (29.6)	21.4 (21.3)	0.002
Max cartilage damage score				0.209
0/1	18.4 (15.1)	0.0 (0.0)	18.4 (16.4)	
2	34.2 (28.0)	1.8 (19.4)	32.4 (28.7)	
3	39.5 (32.4)	2.9 (30.5)	36.7 (32.6)	
4	29.9 (24.5)	4.7 (50.1)	25.2 (22.4)	
Max osteophytes score				0.093
0	6.8 (5.6)	0.0 (0.0)	6.8 (6.1)	
1	52.5 (43.0)	1.8 (19.4)	50.6 (45.0)	
2	49.3 (40.4)	4.5 (47.8)	44.8 (39.8)	
3	13.4 (11.0)	3.1 (32.8)	10.3 (9.2)	
Max BML score				0.758
0	53.3 (43.7)	2.8 (30.4)	50.5 (44.8)	
1	26.6 (21.8)	1.8 (19.5)	24.8 (22.0)	
2	29.5 (24.2)	3.2 (33.9)	26.3 (23.4)	
3	12.5 (10.3)	1.5 (16.2)	11.0 (9.8)	
MRI effusion				0.905
0	31.3 (25.6)	1.7 (17.7)	29.6 (26.3)	
1	54.6 (44.8)	5.1 (54.7)	49.5 (43.9)	
2	30.9 (25.3)	2.1 (22.0)	28.8 (25.6)	
3	5.2 (4.3)	0.5 (5.6)	4.7 (4.2)	

^a^Our analysis is population weighted using decade-gender stratum sampling weights, and the reported numbers are weighted numbers

** Chi-square test for categorical/binary variables, or t-test for continuous variables

All analyses were performed on SAS v9.4 (SAS institute, Cary, North Carolina).

To ensure our results were population-representative, we used a sample weight for the baseline sample according to the proportion of the population sampled for a given age and sex cell compared to proportion of the sample that the given cell made up. The weight of the cell was scaled to sum to the baseline sample size (255). In this study, only 122 participants remained from the original sample of 255. To ensure our results remained population-representative, a sample weight was developed for the current sample size by taking the ratio of the baseline sample proportion in a given age-sex cell over the current sample proportion in that cell, multiplied by the baseline sample weight. The sample weight was scaled to sum to the follow-up sample size (122). All analyses in the present study were weighted with the current sample weight. The discussion of sample weights can be found in Sayre et al. [16].

To ensure our results were not altered by our small sample size we carried out a second sensitivity analysis where we used the data from the 3 year follow up and a separate set of data that was collected between the 3 year follow up to the 7 year follow up. The second data set used the 3 year follow up data as a baseline and compared it to the progression results at the 7 year follow up. With this sensitivity analysis we effectively nearly doubled our sample size to 230 participants. We ran the analysis twice with two different definitions. In the first definition, we ran the sensitivity analysis assuming no non-statin users started a statin during the follow up period, therefore the two data sets had the same number of statin users. In the second definition, we assumed that all the participants who developed dyslipidemia at 3 year follow up were treated with a statin from the 3 year follow up to the 7 year follow up, which would increase the number of statin users in the second data set and in our study. Note that these alternative definitions were necessary because statin use data was not collected at 3 years. In the sensitivity analysis, we adjusted for sex, age, BMI, and our propensity score recomputed using the new statin definition.

## Results

Of 255 participants seen at baseline, 122 completed the seven-year follow-up (Fig. 1). From the initial 255, 40 participants were lost to follow up, 6 were deceased, and 36 declined participation. Of the remaining 173 participants, 51 met the exclusion criteria. Of the 51 excluded, 4 developed a rheumatologic condition, 6 had comorbidities, 16 underwent arthroplasty, 11 moved away, 6 were unable to attend the study center, 4 had MRI contraindications, and 4 had incomplete MRIs [16]. Of the 122 participants, 9.4 (7.7%) were statin users (Table 1). Statins (and dosage) used by participants included atorvastatin (10-40 mg daily), prevastatin (20 mg daily), rosuvastatin (10-20 mg daily) and simvastatin (40-60 mg daily). At baseline the mean age was 55.5 years (SD 9.1), 68.0 (55.7%) were female, and mean BMI was 26.1 (SD 4.0). For diagnosed comorbidities, 18.9 (15.5%) had hypertension, 15.9 (13.1%) had dyslipidemia, 2.7 (2.2%) had cardiovascular disease, 2.3 (1.9) had cerebrovascular disease, and 10.4 (8.5) had diabetes mellitus. Mean WOMAC pain, function, and stiffness scores were 19.1 (SD 17.3), 16.8 (SD 17.3), 23.3 (SD 23.0), respectively. The majority of participants had KL grade 0 (40.8%), while KL grades 1 to 4 were seen in 19.7, 22.5,11.3 and 5.8%, respectively. The characteristics that differed between statin users and non-users were age (61.7 (8.9) and 55.0 (9.0) respectively), BMI (29.5 (4.2) and 25.8 (3.9) respectively), KL grade, and WOMAC stiffness (45.7 (29.6) and 21.4 (21.3) respectively). For KL grade majority of statin users were KL 3 and majority of non-users were KL 0. At 7 year follow up, 60.5% of participants had cartilage damage incidence/progression, 66.6% had osteophyte incidence/progression, 51.5% had BML incidence/progression, and 45.4% had effusion incidence/progression (Table 2).

**Table 2:** Frequency of incidence/progression of MRI features in statin non-users and statin users

	Overall	Statin non-users (*n* = 112.6)	Statin users (*n* = 9.4)	Chi square *p* value
Cartilage damage incidence/progression	73.8 (60.5%)	68.6 (60.9%)	5.2 (55.3%)	0.734
Osteophyte incidence/progression	81.2 (66.6%)	73.2 (65.0%)	8.0 (85.2%)	0.209
BML incidence/progression	62.8 (51.5%)	57.7 (51.2%)	5.1 (54.8%)	0.830
Effusion incidence/progression	55.4 (45.4%)	50.0 (44.4%)	5.4 (57.3%)	0.447

Logistic regression results are shown in Table 3. In analyses, adjusted for age, sex BMI, and propensity score, no statistically significant association of statin use was seen with cartilage damage incidence/progression with an OR of 0.82 (95% CI 0.17–4.06). Similarly, there was no significant association of statin use with incidence/progression of osteophytes (OR 3.48; 95% CI 0.40–30.31), BML (OR 0.61; 95% CI 0.12–3.02), and effusion (OR 2.38; 95% CI 0.42–13.63).

**Table 3:** Association of Statin use with incidence/progression of MRI features

OA incidence/progression definition	Unadjusted OR (95% CI)	Propensity Adjusted OR (95% CI)	Fully adjusted OR (95% CI)
Cartilage score	0.79 (0.21, 3.03)	1.02 (0.23, 4.60)	0.82 (0.17, 4.06)
Osteophyte score	3.09 (0.49, 19.51)	3.87 (0.52, 28.65)	3.48 (0.40, 30.31)
BML score	1.16 (0.30, 4.41)	0.76 (0.17, 3.50)	0.61 (0.12, 3.02)
Effusion progression	1.79 (0.44, 7.26)	3.55 (0.66, 19.19)	2.38 (0.42, 13.63)

*OR* Odds ratio, *BML* Bone marrow lesion

Propensity Adjusted OR: adjusted for propensity score only

Fully adjusted OR: adjusted for propensity score, age, sex, BMI

Propensity score model: Statin use ~ hypertension, dyslipidemia, cardiovascular or cerebrovascular disease, diabetes

As in the primary analysis, the results of both sensitivity analyses were not statistically significant, regardless of adjustment for KL grade and statin use definition at 3 years.

## Discussion

The purpose of this study was to evaluate whether the use of statins slows OA progression as proposed in recent studies. Several previous studies found a relationship between statin use and OA progression, however, currently there is no consensus on the topic. Additionally, of all the studies that have looked at OA progression none have used MRI to evaluate progression. Our study showed that statins had no effect on cartilage damage, BML, osteophyte, and effusion incidence/progression on MRI in a cohort with knee pain and predominantly pre-radiographic OA. To our knowledge, this is the first study to evaluate statin use in relation to MRI outcomes of pre-radiographic knee OA.

There were a few studies that came to different conclusions than our own and found an association between statin use and OA progression [9–11, 20]. In an observational study by Clockaerts et al [10], statin use was associated with reduced progression of knee osteoarthritis using KL grade to assess for radiographic progression of OA. Clockarts et al. [10] used a cohort where most of the participants had KL grade less than 2. They found that statins had no effect on hip OA whereas it did reduce progression in knee OA, the authors hypothesized that this may be due to different mechanisms of progression for the two joints [10]. This study used straight leg radiographs to assess for knee OA progression. This method is not as reliable in assessing joint space width when compared to radiographs with 20–30 degree knee flexion [10]. The reliance on straight leg radiographs could reduce the accuracy of their measurements and conclusions. In another study by Beattie et al. [11], statins were found to modestly decrease the risk of radiographic hip OA progression (using a modified Croft grade), although these results were not significant. This study only included elderly white women which reduces the generalizability of the conclusions drawn from the study. Additionally, this study looked exclusively at hip OA which may not relate to knee OA in terms of progression as hypothesized by Clockarts et al. Haj-Mirzaian et al [12], conducted a longitudinal analysis of the data from the OAI study. Hag- Mirzaian looked specifically at a subset of participants with knee OA and Heberden nodes (HN). They found that statin use in participants with knee OA and 2 or more HN reduced the risk of JSN progression [12]. However, this benefit was not seen in the individuals with less than 2 HN [12]. In contrast to these three studies, Eymard et al [9] found that statin use was associated with increased JSN over the 3 years follow up period when compared to non-users. Eymard et al’s [9] cohort only used caucasian participants with a KL grade of 2 or more. Like our study, Eymard et al. [9] only assessed statin use at baseline and did not assess duration which is a limitation in their methodology. Furthermore, this is not a representative cohort since it is limited to caucasian participants with radiographic OA.

Two studies carried out by Riddle et al [14] and Veronese et al [13] evaluated the association between statin duration and OA progression. Like our study, both studies found no association between statin use and progression of OA. Riddle et al. [14] conducted a longitudinal study where the mean KL grade was calculated each year for a four-year period in a cohort of participants with radiographic OA in at least one knee. The cohort was obtained from the Osteoarthritis Initiative database (OAI). The participants also indicated their statin usage each year by bringing in their current medications. They found that there was no significant change in the mean KL grade for statin users and non-users. Veronese et al. conducted a longitudinal cohort study where they assessed how duration of statin use affected the KL grade of OA for each participant over a 4 year follow up period. They also obtained their cohort from the OAI database. The cohort included participants if they had OA with knee pain for 30 days or were at high risk of developing knee OA. Duration of statin use was assessed by interviewing participants on the use of several statins at baseline and at follow up and the responses were recorded. They found no association between statin duration and radiographic progression (increased KL grade) of OA. Both studies assessed duration of statin use, which was not assessed in our study, and came to the same conclusion as we did. However, as these two studies only included participants with radiographic OA or suspected radiographic OA, their results do not take into account those with pre-radiographic OA.

This study is subject to its own limitations. Firstly, our two cohorts differed in terms of KL grade distribution which could have confounded our results. However, we ran our primary analysis while adjusting for KL grade and this did not change the significance of the results or the conclusions of our study. Secondly, our study only assessed statin use at baseline, and we did not assess continuation of statin therapy for the duration of the study. However, when a patient is started on statin therapy they tend to be on it for life, and it is unlikely that the statin will be discontinued. Additionally, with our sensitivity analysis we showed that even if we assume that all those with a new dyslipidemia diagnosis at 3 year follow up will be starting a statin, our conclusions do not change. The effect of statins on OA progression may be dose dependent and this may explain the lack of association observed in our study. Since participants in our study would have been dosed according to their LDL cholesterol level and target levels, they may have been dosed inadequately to slow OA progression. However, similar to our study, previous cohort studies would have had participant’s statins dosed according to the comorbidity it was indicated for. Therefore, these studies would have found a correlation with similar statin dosing in our patients. It is possible that duration of statin use can affect OA progression as well. Although duration was not assessed in our study, it was shown to not influence OA progression by Riddle et al. [14] and Veronese et al. [13]. Additionally, even if the statin users in our cohort started statin therapy immediately prior to enrollment they would have 7 years of statin therapy at follow up which would be enough time to see any potential effects on OA incidence/progression. Lastly, our study did not assess the effect of different statins on OA progression. Veronese et al. [13] found that atorvastatin was shown to have a lower risk of pain worsening and rosuvastatin was associated with a higher risk of pain worsening. Although our study did not look at pain progression, different statins could have different effects on cartilage, osteophyte, BML and effusion progression on MRI. Our study did not evaluate different statins due to sample size limitations. This should be evaluated further in future research.

Our study has two major strengths: the use of MRI and a population-based cohort. As stated previously, a major limitation of the current literature is that previous studies used radiographic outcomes to determine OA progression. Radiographs cannot show early OA changes that can be seen on MRI and may result in inaccurate measures of OA progression. In addition to the use of MRI in this study, we also used a population-based cohort including mostly pre-radiographic but also radiographic stages of disease making our results applicable to either group of OA patients. Lastly, our cohort only had individuals with knee pain. Individuals with knee pain predominantly make up the population that seeks care for their OA and therefore the outcomes in this population are of greater clinical interest.

## Conclusion

In this population-based cohort of participants with knee pain and predominantly pre-radiographic OA, statin use was not associated with incidence/progression of cartilage damage, osteophytes, bone marrow lesions, and knee effusion on MRI after 7 years of follow up. As such, our results show that statins are not harmful or beneficial to those with pre-radiographic knee OA.

## Data Availability

The datasets used and/or analysed during the current study are available from the corresponding author on reasonable request.

## Abbreviations

OA: Osteoarthritis
MRI: Magnetic resonance imaging
BML: Bone marrow lesions
BMI: Body mass index
NSAID: Nonsteroidal anti-inflammatory drugs
IL-1B: Interleukin-1B
MMP: Matrix metalloproteases
NO: Nitric oxide
JSN: Joint space narrowing
VALSEKO: Vancouver Longitudinal Study of Early Knee Osteoarthritis
WOMAC: Western Ontario and McMaster Universities Osteoarthritis Index version VA 3.1
KL: Kellgren Lawrence
SPGE: Spoiled gradient echo
FSE: fast spin-echo
MOAKS: MRI osteoarthritis knee score
SD: Standard deviation
OR: Odds ratio
HN: Heberden nodes
OAI: Osteoarthritis Initiative database
